# Sexual activity and functioning after breast cancer treatment: perspectives on the importance of pleasure from a radiotherapy cohort

**DOI:** 10.1186/s12905-025-04063-w

**Published:** 2025-10-29

**Authors:** Melanie Besculides, Lauren Carney, Ksenia Gorbenko, Sheryl Green, Melissa Brito, Jezelle Lynch, Carly Feldman, Cindy Munoz, Madhu Mazumdar, Deborah C. Marshall

**Affiliations:** 1 Institute for Healthcare Delivery Science, Mount Sinai Health System, New York, NY USA; 2 Department of Population Health Science and Policy, Icahn School of Medicine at Mount Sinai, New York, NY USA; 3 Department of Radiation Oncology, Icahn School of Medicine at Mount Sinai, New York, NY USA; 4 Sidney Kimmel Medical College, Philadelphia, PA USA

**Keywords:** Sexual health, Pleasure, Quality of life, Breast cancer, Radiotherapy

## Abstract

**Background:**

Breast cancer affects millions of women and while treatment can be lifesaving, it also has numerous side effects, including those related to sexual function, which often impact the ability to experience pleasure from sex. This study aimed to understand how breast cancer survivors define sexual activity, identify important aspects, and ascertain perceived impacts of treatment on sexual activity and function, particularly related to pleasure.

**Methods:**

Semi-structured interviews were conducted with women who were breast cancer survivors at least one-year post-radiation. Priority populations were oversampled (racial/ethnic/sexual/gender minorities, those aged over 65 or under 45). The sample included 23 participants: 11 Non-Hispanic White, 8 Non-Hispanic Black, 2 Non-Hispanic Asian, 2 Hispanic/Latinx, and 1 transgender participant. Mean age was 58.6 years (range 34–77, SD = 12.2).

**Results:**

Four themes were identified: 1) *Sexual activity is defined broadly*,* and intimacy and pleasure are key.* Participants defined sex in broad terms and highlighted the importance of being close with partners and pleasure. Sex was important across the lifespan and among participants from different backgrounds, 2) *Few anticipated sexual side effects*: Although treatment side effects were common, few expected them, 3) *Clinician communication is lacking or mistimed*: Participants did not remember conversations about potential side effects because they did not happen, or they were not in a mental space to process them, 4) *Sexual health side effects are plentiful and for many*,* long-lasting*. Changes to breast sensitivity and ability to achieve orgasm were reported. Breast cancer survivors conceptualized sexual activity through a broad lens and highlighted the importance of sex, particularly pleasure and intimacy. Participants reported a variety of negative effects from treatment that impacted pleasure, and patient/clinician communication on sexual health was perceived to be lacking.

**Conclusion:**

Clinicians may improve survivors’ quality of life by acknowledging clinical origins of sexual dysfunction, prioritizing pleasure and intimacy in the development of interventions to prevent or mitigate sexual side effects and fostering open communication.

**Supplementary Information:**

The online version contains supplementary material available at 10.1186/s12905-025-04063-w.

## Background

Breast cancer is the most prevalent malignancy in the United States, with an estimated 4.1 million female survivors [1]. In 2024 there were 310,720 new cases of invasive breast cancer diagnosed [2]. Standard breast cancer treatment includes a combination of surgery (including lumpectomy or mastectomy), radiation, and systemic therapies including chemotherapy, targeted or immuno-therapy and endocrine therapy [3]. These lifesaving treatments have numerous side effects, including those related to sexual function (e.g., vulvovaginal changes due to endocrine treatments impacting the vagina and erectile tissues and loss of breast sensitivity), which often impact the ability to experience pleasure from sex [4–6]. Sexual health is a key component of the many aspects of quality of life (QOL) impacted by cancer treatment and is increasingly important as survival rates continue to improve [5, 7]. Poor sexual health can affect emotional well-being, relationships, self-image, physical health and social well-being [8]. Sexual functioning is a complex biopsychosocial phenomenon, and there are specific opportunities to intervene within the cancer care continuum through both the biological and psychosocial lens. However, our current understanding of the biological effects of cancer treatments on sexual pleasure in women and their long-term impacts on sexual function is extremely sparse due to longstanding historical bias.

To develop effective interventions to address women’s priorities for sexual functioning, we sought to investigate the impact of cancer treatment on women’s sexual pleasure and the importance of pleasure in sexual health for breast cancer survivors. We focused on breast cancer because treatment can directly affect the sexual organs that are responsible for pleasure and because historically any discussion around sexual health for this population has focused on fertility and menopause rather than pleasure [9]. We conducted a qualitative investigation to elucidate key characteristics of sexual activity, sexual outcomes, communication, and preferences related to sexual activity in priority populations. Specifically, we sought to detail breast cancer survivor’s lived experiences, including how people define sexual activity, its importance, communication with providers about sexual side effects of treatment, and the impact of treatment on sexual activity and function, including sexual pleasure.

## Methods

The Multiphase Optimization Strategy (MOSt) [10] framework for intervention development guided the study method selection. Our methods correspond with the preparation phase, which lays the groundwork for the development of a conceptual model of change. The development of our conceptual model of change is based in biopsychosocial models of sexuality and sexual response [11, 12], and we used a qualitative descriptive approach to identify the importance of and factors that impact pleasure post-cancer treatment to inform intervention design. This article follows the COREQ reporting standards (Consolidated Criteria for Reporting Qualitative Research) [13]. 

Patients were recruited to participate in a prospective cohort study by radiation oncology clinicians during routine follow-up visits from September 2022 to February 2024. Consented participants completed study surveys post-visit on a tablet or were contacted by a study team member for later participation. All participants were older than 18, female or gender-diverse, English-speaking, had an Eastern Cooperative Oncology Group (ECOG)^1^ performance status less than 3, were able to complete an electronic survey, had completed local cancer treatment at least one year prior, and agreed to be contacted for and were willing to sign a written informed consent and participate in qualitative interviews. Participants self-identified their race, ethnicity, sex assigned at birth, gender identity, and sexual orientation on the survey. As part of the study consent, participants were asked if they were interested in being interviewed for a qualitative study. Among those interested, participants were contacted by the qualitative team via email and/or by phone and interviews were completed among those willing to participate. Understudied priority populations, including racial/ethnic minorities, sexual and gender minorities, participants older than 65 years and younger than 45 years were purposively sampled for interviews. Sample size was determined by thematic saturation. Participants who completed the interview were sent a $30 Amazon gift card via email as compensation for their time.

All interviews were conducted in English using a semi-structured guide (Appendix A) and lasted approximately 30 min. After the first three interviews were conducted the qualitative team met to revise the interview guide to ensure questions gathered the desired information. Interviews were conducted by three researchers with significant qualitative experience and doctoral-level training in behavioral science (MB), medical sociology (KG), and clinical psychology (LC). The researchers were all female and not involved in the cancer care of the participants interviewed. Before commencing the interview, the interviewer introduced themselves and explained the purpose of the study. This information was also provided by email at the time of scheduling. Interviews were conducted via HIPAA-compliant video conferencing software (Zoom Video Communications) and recorded with permission. Recordings were transcribed professionally and checked for accuracy. Transcripts were imported into Dedoose 9.0 for coding and analysis. The first two interviews were independently reviewed, annotated, and open coded by the three-person qualitative team to develop a preliminary code book. Transcripts were then coded independently by two team members, and weekly team meetings were held to discuss any differences in coding and emerging codes until the codebook was stable (i.e., no new codes were found). Saturation of knowledge was also discussed at these meetings and we interviewed until we had representation from all priority populations. The final code book (Appendix B) was then applied to all previously coded transcripts and iterative thematic analysis was conducted [14]. Iterative thematic analysis is a dynamic and flexible process of qualitative data analysis, in which researchers continuously revisit the data, codes, and themes, refining them progressively as their understanding deepens.

Qualitative rigor was maintained through the following strategies: [15]

- Credibility: We employed analyst triangulation by involving researchers from diverse disciplinary backgrounds. Regular team meetings incorporated structured reflexivity exercises to critically examine and mitigate potential biases in data interpretation.
- Transferability: We provided a contextualized description of the research setting and detailed our sampling strategy to enable readers to assess the applicability of our findings to other contexts.
- Dependability: We maintained a comprehensive audit trail documenting each step of data collection and analysis, including any procedural changes, to ensure methodological transparency and reproducibility.
- Confirmability: Our team of experts systematically reviewed all preliminary and final findings to evaluate the validity, coherence, and clinical relevance of our interpretations.

This study was conducted at three clinical sites in the Mount Sinai Health System and was approved by the Institutional Review Board (#22–00127).

## Results

Ninety-one participants (81% of the overall study) consented to being contacted to participate in an interview. Of these, 36 were contacted for an interview and 23 completed interviews. Table 1 shows the demographic characteristics of the population interviewed. Most (*n* = 15) of the sample were aged 45–69 years (range 34–77, mean = 58.6, standard deviation = 12.2). Thirteen of participants were partnered, all identified as women, one was assigned male sex at birth, and most of the sample (*n* = 22) reported their sexual orientation as straight, with one participant responding with “prefer not to answer.” The sample included 11 Non-Hispanic White, 8 Non-Hispanic Black or African American, 2 Hispanic/Latino Black or African American, and 2 Non-Hispanic Asian participants. Most (*n* = 18) of the sample reported being sexually active in the last 30 days. Twenty participants underwent lumpectomy, 15 received endocrine therapy, and 8 received systemic therapy.

**Table 1:** Characteristics of qualitative interview sample of participants with breast cancer

Characteristic	*n* (%), Total *N* = 23 Women
Age category at survey, years	
18–44	3 (13%)
45–69	15 (65%)
70+	5 (22%)
Marital Status	
Non-partnered	10 (43%)
Partnered	13 (57%)
Sex at birth	
Female	22 (96%)
Male	1 (4.3%)
Sexual Orientation	
Prefer not to answer	1 (4.3%)
Straight	22 (96%)
Race/Ethnicity	
Hispanic or Latinx Black	2 (8.7%)
Non-Hispanic Black	8 (35%)
Non-Hispanic White	11 (48%)
Non-Hispanic Asian	2 (8.7%)
Sexually Active (last 30 days)	18 (78%)
Surgery Type	
Lumpectomy	20 (87%)
Mastectomy	3 (13%)
Endocrine (Hormone) Therapy	15 (65%)
Systemic Therapy (Chemotherapy)	8 (35%)

Four themes were identified: 1) Sexual activity is defined broadly and intimacy and pleasure are key; 2) Few anticipate sexual side effects; 3) Communication is lacking or mistimed; and 4) Sexual health side effects are plentiful and for many, long-lasting.

### Theme 1: sexual activity is defined broadly, and intimacy and pleasure are key

Most participants had broad definitions of sexual activity. This was true regardless of age and race/ethnicity. For many participants, oral sex and masturbation were part of their definition and for a few, anal intercourse was included.

*“Any kind of penetration*,* either vaginal*,* oral*,* anal*,* as well as stimulation*,* either with hands*,* or toys” [age 70+*,* Black NH]*.

*“Intercourse*,* foreplay*,* orgasms*,* masturbation.” [age 70+*,* White NH]*.

Physical and emotional closeness, which many termed intimacy, was mentioned repeatedly in definitions of sexual activity. This included cuddling, kissing, touching, and connecting with a partner on an emotional level.

*“Being with another person*,* like being with my husband*,* just being…having intercourse with him*,* and sensual touching*,* and maybe sometimes just hugging*,* you know*,* just having the body*,* the feel of the body next to somebody else*,* you know*,* that was important to me.” [age 45–69*,* Black NH]*.

*“I think it’s everything from just you know touch and hugging and kissing to you know to intercourse.” [age 45–69*,* White NH]*.

Sexual activity was an important aspect of life for many participants, regardless of age and race/ethnicity. Although some noted a decline in interest and frequency over time with age and lack of available sexual partners, sexual activity was still important for many people, even without partners. Intimacy and touching were often mentioned as the most important aspects of sexual activity; participants noted the need to feel connected emotionally to their partners, not just physically.

*“…not just having intercourse. Sexual activity for me is showing my husband how I feel about him. My emotions. I love him. It’s not just the act of just the sex.” [age 45–69*,* Black NH]*.

Pleasure was also very important to many participants. Nipple and clitoral stimulation were discussed as being pleasurable and achieving orgasm for self-and/or partner was mentioned frequently as being important.

*“.it was very important to me that we were connecting both on the intimate level*,* giving each other orgasms*,* and also just connecting physically*,* whether*,* you know*,* on any level*,* whether*,* like I said*,* snuggling or all the way through to sexual intercourse*,* you know*,* and every degree in between.’ [age 18–44*,* White NH]*.

*“I really want to make sure my husband is pleased more than… I mean*,* I’d love to get pleased myself*,* but it doesn’t always happen. But I know that it’s more regularly that I can make sure that he comes to orgasm*,* whereas I don’t always. ….So having an intimate relationship with him so that we are both happy is important. But ideally*,* if we could both orgasm*,* that would be better. It just isn’t reality….I think the emotional part is tied with pleasing my partner*,* so I think that that’s heavily in there.” [age 45–69*,* White NH]*.

### Theme 2: few anticipate sexual side effects

The overwhelming majority of participants were not aware of how their cancer treatment would affect their sexual function before treatment began.

*“I wasn’t concerned because I didn’t really know. I didn’t know if it would affect me at all… I was surprised when I was diagnosed*,* so before that I had no*,* you know*,* real knowledge of how that would affect me sexually. That didn’t cross my mind at that point.” [age 45–69*,* Asian]*.

Even if they were aware, they reported being more focused on treatment and survival.

*“You know*,* I was so preoccupied with trying to figure out what kind of cancer it was and how to treat it that I really didn’t think about sexual health at all. To me that was just so not on the front burner for me. [age 45–69*,* White NH]*

For those who had concerns before treatment, a couple were worried about burns from radiation, how their bodies would change, and how this would affect their partner relationships.

*“I don’t think I was that concerned. I think I was concerned about you know what I would look like or how it would affect my appearance and how might that affect my relationship. But you know as far as my own sexual health I wasn’t that concerned and I was just wondering the impact on our relationship.” [age 45–69*,* White NH]*.

Among the handful of participants who had concerns about sexual health before treatment, the potential for vaginal dryness and loss of breast sensitivity were mentioned. Only one breast cancer survivor stated they were very concerned about how treatment might affect their sexual health.

### Theme 3: clinician communication is lacking or mistimed

Participants reported that clinician communication about sexual side effects is either lacking or mistimed and that aspects of identity affect what is communicated.

#### Subtheme 3a. nothing was said or I wasn’t ready to hear it

Almost no one remembered clinicians discussing potential sexual side effects of treatment with them. Some were certain that discussions about side effects did not occur while others noted that issues may have been raised but they were not in a headspace to process them, or they were not sexually active, so the discussion did not apply to them.

“*I won’t say no*,* but I don’t even really remember*,* because when you go in and they tell you we have good news. We have bad news. For some reason everything you start hating everything from a far…So I don’t want to lie and say they didn’t. But I can’t remember. [age 45–69*,* Black NH]*

Among the few participants who had concerns about sexual health, none reported discussing these concerns with their clinicians before treatment.

#### Subtheme 3b. identity is perceived to affect communication

Some participants perceived that aspects of their identity such as race and age affected conversations about sexual health. For instance, one patient who was Black believed she received less information about the sexual side effects of cancer treatment because of her race.

*“I don’t believe that they consciously didn’t tell me*,* but I believe because I’m an African American that somewhere in the back of their mind they didn’t feel it was important to tell me. I’m an African American woman*,* and a lot of times we get less information*,* we get less care.” [age 45–69*,* Black NH]*.

Another participant felt clinicians treated her as less important because she was older.

*“I don’t know. I think it’s more like age because I find out that I’m experiencing more discrimination of age than the gender or race. Being a older woman it look like you passed your prime*,* you know*,* it’s like your life is not as important as it should be. So it’s like you’re a second thought. And I find that happen a lot.” [age 70+*,* Black NH]*.

Two participants who were younger noted that a lot of attention was given to whether they wanted to freeze their eggs so future children might be possible. One of these women noted that being a feminist, she was bothered by the sole focus on childbearing with no attention to other aspects of sexual health.

### Theme 4: sexual health side effects are plentiful and for many, long-lasting

Many participants reported sexual side effects including changes to desire, vaginal dryness, and breast sensation and the changes were long lasting for many women.

#### Subtheme 4a. desire is diminished but sex is still important

Changes in sexual desire and sexual function were reported by many participants after treatment. For many, desire decreased and did not return. Participants often reported that they continued to engage in sexual activity despite lack of desire because it was important in their relationships.

*“I really don’t have any desire to have sex*,* but I know it’s important in my relationship with my husband*,* so I do it more as a mutual*,* you know*,* pleasure thing. I mean*,* it’s not unpleasant to have sex*,* it’s just I don’t feel hungry for it*,* you know what I’m saying? It’s like eating because you have to eat*,* because it’s good for you*,* not because you’re hungry.” [age 45–69*,* White NH]*.

Some participants partially attributed decreases in sexual desire to age, some were scared or traumatized from the cancer, while others noted that changes to lubrication and breast sensitivity affected desire.

*“I had to recover from this traumatic experience*,* and I just wasn’t feeling sexual or didn’t want to be bothered.” [age 70+*,* Black NH]*.

#### Subtheme 4b. dryness is painful and bodily changes are embarrassing

Vaginal dryness was a commonly reported symptom. For some, dryness existed before treatment, which many associated with menopause. For others, dryness was new, particularly among those who were undergoing endocrine therapy. Some women engaged in intercourse despite lubrication issues to have intimacy.

*“But most of the time it was very uncomfortable and it hurt a lot*,* as opposed to before. And I think it’s because of the dryness that I have been experiencing. But it was just very uncomfortable*,* very unsatisfying. But I did it for the emotional intimacy.” [age 18–44*,* Black NH]*.

Participants who reported dryness had often found treatments either on their own or with guidance from a clinician that helped. These include lubricants, moisturizers, and vaginal laser treatment. One participant who was younger reported being embarrassed and defeated about needing to use lubrication.

*“It also manifests in having a difficult time with lubrication*,* feeling embarrassed about that*,* feeling ashamed about that*,* especially being younger.” [age 18–44*,* White NH]*.

Participants also reported covering up their bodies in front of their partners to hide uneven breasts.

*“…like what I didn’t always get totally undressed. So let’s put it that way*,* to camouflage the fact that my breast were uneven.” [age 70+*,* Black NH]*.

#### Subtheme 4c. breast sensation-a hotspot no longer

Many participants who underwent lumpectomy reported changes to breast sensation. Some stated that breasts were overly sensitive and sore, while others reported a loss of breast sensation and ability to stimulate the nipple.

*“It’s like the sensation of the nipple*,* it’s a little different like. I would say that was like one of my hot spots*,* if it’s okay to say that*,* like that was like a trigger for me. It’s still a trigger*,* but is not so that*,* its not the way it was*,* you know? [age 45–69*,* Black NH]*

Participants reported either a complete loss of sensation or a decrease in the intensity of the sensation. Increases and decreases to sensation affected both breasts in some cases, not just the breast that underwent surgery. Changes to breast sensation affected the desire to be touched and the ability to derive pleasure from sexual activity.

*“My husband couldn’t touch my breast. It couldn’t be*,* it couldn’t even be the right one like every time he comes too close to it. To me it was a turn off it’s like*,* I don’t want you to see that I don’t want you to touch it.” [age 45–69*,* Black NH]*.

Although some participants reported that the changes to sensation improved or resolved over time, this was not true for many participants, and they believed sensation may never return. Although changes to breast sensation were common, few discussed these changes with their clinicians.

#### Subtheme 4d. pleasure is a problem

Participants also reported other physical changes that impacted their ability to become fully aroused and have pleasurable sexual experiences. Vaginal tightness, being irritated by touch, mood swings, hot flashes, exhaustion, and changes to the appearance of the breasts that made participants self-conscious were all mentioned. Several participants noted that achieving orgasm was more difficult after treatment even with self-stimulation.

*“There’s like less sensation…even in self-stimulation it’s just like… I’m going to use this term—it really doesn’t do it for me. Like there’s…it doesn’t feel like there’s anything in the area that is… I don’t want to say I can’t really feel anything*,* but it kind of feels just like numb*,* a little bit. [age 18–44*,* Black NH]*

*“I don’t have that desire. I don’t have… And I can’t*,* even with masturbation*,* I can’t get to orgasm*,* so it doesn’t pay*,* you know*,* it’s just… So I’ve just… Because one day I said eh*,* let me try it and see what happens. Nothing.” [age 45–69*,* Black NH]*.

*“But it doesn’t mean that I don’t get some pleasure from it. I do get pleasure from it. It’s just not the intensity of an orgasm. But I do occasionally have orgasms. Usually they have to be stimulated with a vibrator afterwards. And sometimes it works and it’s great and sometimes it doesn’t work and I get sore and it hurts*,* and I give up”. [age 45–69*,* White NH]*

It was rare that participants discussed these issues with their clinicians. Some stated that sex is not a comfortable topic of discussion for cultural reasons, and a few noted that they may have discussed problems if clinicians had asked questions about sexual side effects.

## Discussion

This qualitative study found that sexual activity and pleasure were important for a diverse sample of breast cancer survivors, including survivors who were older and/or unpartnered. Many experienced unexpected cancer treatment side effects that impacted their ability to have pleasurable sexual experiences. Participants also reported a lack of communication with clinicians regarding the potential for sexual side effects.

This study revealed the multitude of ways that sex was defined by our participants. This knowledge can help clinicians select appropriate language for conversations about sexual health. For example, they can offer a broad definition of sexual activity that includes vaginal or anal intercourse, oral sex, masturbation (with or without a partner), or anything else that the patient might consider sexual activity.

The study also found that sex is important across the lifespan and for those from different backgrounds and with different identities. Previous research has shown that it is common for women with cancer who are older to continue to be sexually active and that age and health status are barriers to clinician communication about sexual health. Some of the participants in our sample perceived that aspects of their identity affected the information they received from clinicians. This is an area that requires prioritization so long-standing disparities are not exacerbated. Discussion about sexual health should occur with all patients, not just those thought to be sexually active.

Many participants reported difficulties with pleasurable sensation, arousal and orgasm due to both hormone-related symptoms such as vaginal tightness and changes to breast integrity including loss of sensation, scars and other surgical changes. The changes reported affect each phase of the sexual response cycle (excitement, plateau, orgasm, resolution) described by Masters and Johnson, highlighting the multifaceted nature of sexual changes post-cancer treatment [16]. Participants often continued to engage in sexual activity despite issues because it was important to their relationships. Future research may consider the role sexual equity theory plays in navigating sexual difficulties within relationships, as sexual side effects can create inequities in sexual satisfaction between partners which can cause distress [17].

Efforts can be made regardless to improve sexual encounters for patients experiencing dysfunction who choose to continue to engage in sexual activity. For instance, discussing the use of lubricants or sexual aids with patients may improve sexual experiences because their use can reduce discomfort, increase arousal, and improve pleasurable sensation. Research has also shown that nipple stimulation causes or enhances arousal for the vast majority of women [18] and patients who have undergone mastectomy experience more sexual dysfunction (desire, arousal, orgasm) than patients undergoing breast-conserving procedures due to changes in breast sensation [19]. Researchers who understand the importance of nipple-areolar sensation in arousal and orgasm have begun to test the use of bionics to restore breast sensation [20]. Even without such technology, clinicians can acknowledge that sexual dysfunction can have treatment-related causes (radiation, endocrine therapy, systemic therapy, or combinations of these) so that patients understand what is happening to their bodies and feel validated that their symptoms are real and have clinical roots. Finally, our research showed that intimacy is an important aspect of sex and, consistent with previous research, patients engage in sexual behavior even when they lack desire because they feel it is important to their relationships [21]. Discussions may include a referral to a sexual health counselor to help foster patient/partner discussions around feelings of embarrassment due to physical changes in their bodies such as vaginal dryness or uneven breasts.

Most participants did not recall clinicians discussing the potential for sexual side effects, either because these conversations did not occur or because participants were not in a mental space to process them. This finding is consistent with previous qualitative research on pelvic cancer survivors who experienced distressing sexual health treatment side effects for which they felt unprepared by clinicians [22]. Additionally, one study found that less than a third of patients with breast cancer report ever receiving information about sexual health from a clinician [23] and another found that clinician communication was uncommon even among those who reported sexual problems [24]. Participants believed they were not always capable of processing information about sexual side effects, which could be explained by high emotional stress impacting memory formation during medical appointments [25]. Therefore, clinicians should offer multiple opportunities for discussion and provide informational handouts with contact information before, during, and after treatment. Research has also identified many reasons why clinicians do not discuss sexual health related treatment side effects with female patients with cancer even when they suspect they will occur including clinicians not believing such discussions are their responsibility, lack of knowledge and experience, embarrassment, and lack of resources to provide support if needed [26]. Increased training in how to discuss sexual side effects with breast cancer survivors may improve clinician’s knowledge and comfort with these discussions.

We also found that even when participants had concerns or experienced sexual health side effects, they rarely share them with their clinicians. This is consistent with previous research that patients with breast cancer feel too intimidated or embarrassed to raise sexual concerns but would like clinicians to offer guidance [23]. Research also shows that patients are more likely to discuss sexual issues with clinicians they perceive to be of similar age or sex/gender [26]. While it may not always be feasible to match patients with demographically-matched clinicians, clinicians can initiate conversations by asking open-ended questions about sexual health and treatment side effects in a supportive and non-judgmental manner. Clinicians should also be able to refer patients to sexual health specialists for further care. To ensure patients receive the best possible care, it is also recommended that they address questions about sexual health with any of their clinicians, including surgeons, radiation oncologists, oncologists, primary care doctors, or gynecologists. Efforts focused on empowering patients to take an active role in their healthcare and seek the support they need may also help improve sexual health communication.

### Limitations/strengths

Although participants were selected from multiple practice settings, they were all from one academic institution in an urban setting and the vast majority identified as straight. Interviews were also only conducted in English. A major strength of our study is the diversity we were able to achieve in terms of race/ethnicity, age, and sex at birth.

## Conclusion

This study provides important insights into the lived experiences of breast cancer survivors related to sexual health and function. We found that our sample held broad conceptualizations of sexual activity and found pleasure and intimacy to be key aspects of sexual activity. Participants reported that breast cancer treatment impacted their sexual pleasure, and they perceived a lack of communication from their clinicians about sexual side effects. Clinicians should prioritize the sexual pleasure and intimacy of their patients and communicate about sexual side effects before, during, and after cancer treatment. Patients should be encouraged to ask their oncology clinicians questions about sexual health and explore the expanding range of solutions available to them. Understanding how sexual health is conceptualized, its importance, and the impact treatment has on sexual health and pleasure can help guide future intervention development to improve breast cancer survivors’ sexual and overall QOL.

## Supplementary Information

Supplementary Material 1.

Supplementary Material 2.

## Data Availability

The datasets used and/or analyzed during the current study are available from the corresponding author on reasonable request.

## Footnotes

1 ECOG is a widely used scale that assesses a patient’s functional ability and helps determine appropriate treatment and prognosis.
