# Supplementary material for: Sexual activity and functioning after breast cancer treatment: perspectives on the importance of pleasure from a radiotherapy cohort
Source: BMC Womens Health. 2025 Oct 29;25:522. doi: 10.1186/s12905-025-04063-w (PMC12570742; doi:10.1186/s12905-025-04063-w)
Supplement: Supplementary file 1 — Supplementary Material 1. [file 12905_2025_4063_MOESM1_ESM.docx]

Dedoose Codes Export for Project: RetroSTAR

| **Code** | **Description** |
| --- | --- |
| Accidents | Any issues with incontinence after treatment |
| Advice to others | Any advice patient offers to others about treatment, support, etc. |
| Alone in experience/misunderstood | Patient reports feeling alone or misunderstood after treatment |
| Breast Sensation | Patient reports loss/changes to breast sensation. Excludes sensation in other parts of the body. |
| Concern reporting | Initiating or failing to initiate discussion about concerns before treatment |
| Concerns before | Any sexual health issues patient was concerned about before/during treatment (e.g., how treatment might affect ability to have orgasm) |
| Digital interventions | Preference for a digital format for psych intervention |
| Dilators/aids | Experience with dilators/sexual aids yes or no experience |
| Dryness/lubrication | Issues with dryness or lubrication |
| Fertility preservation | Mention of desire/importance of preserving fertility |
| Goals of treatment | Mention of the importance or discussion of goals of treatment between patient and provider |
| Great quote | Any good quotes we may include in a report |
| Healthcare worker | Patient reports being a healthcare worker and how this might affect knowledge or interaction with providers |
| Identity | Mention of gender, race, age, etc. that patient perceived could affect provider discussion or treatment |
| Impact length | Length of side effects (e.g., still present?) |
| Impact on desire | Treatment impacted desire to be sexually active |
| Impact on enjoyment/pleasure/satisfaction | Treatment impacted enjoyment/pleasure/satisfaction/ability to achieve orgasm with sexual activity |
| Impact on relationships | Treatment impacted relationships |
| Insurance | Mention of importance of, lack of, trouble with insurance |
| Lubricants/Moisturizers | Lubricant/moisturizers used as treatment |
| Needed information | Any information that would have been helpful before, during, after treatment, now |
| Other patients | Discussion of the experiences of other patients with cancer (family, friends) |
| Other physical symptoms | Any physical symptoms reported, excluding physical symptoms that patient clearly connects to sexual health and psychological symptoms |
| Partner support | Patient mentions receipt of support from partner |
| Physical Therapy | Patient reports having received PT for symptoms. Excludes vaginal rejuvenation/ablation |
| Provider discussion | Any discussion/information providers gave to patient before treatment about possible sexual side-effects of treatment (includes content) |
| Provider discussion: format | The format of information providers used (e.g., conversation, printed materials) |
| Provider discussion: initiation | Person that initiated discussion about potential side effects (e.g., provider, patient) |
| Provider discussion: preferences | Preferences/desires for provider discussions. Includes advice to doctors. |
| Provider discussion: quality/satisfaction | Patient assessment of information they received from provider |
| Provider discussion: who | Type of provider who patient had discussion with (e.g., surgeon, oncologist, radiologist, nurse, etc.) |
| Provider next visit | Approximate date of next visit with provider (potential to discussion side effects) |
| Provider relationship | Comfort level in contacting/talking to provider |
| Provider selection | Any mention of ways they choose a provider (e.g., gender, best doctor, recommendations) |
| Psych interventions: experience | Any patient experiences with psychological interventions after treatment |
| Psych interventions: openness | Whether the patient would be open to psych interventions if they were available |
| Psych interventions: preferred format | What format would patient like for psych intervention (e.g., in person, digital) |
| Psychological impact | Psychological side effects of treatment (e.g., depression, in a funk, denial) |
| Resilience/inner strength | Patient discusses drive to survive and be resilient |
| Resources | Mention of coordinated resources (e.g., MSK) |
| Scared/survival | Patient mentions being afraid of dying. Might not have heard what was said/processed b/c of fear. Priority on treatment/survival over sexual function. |
| Searching for information | Patient reports looking for information about side effects or treatment (articles, online, FB group, etc.) |
| Self-advocate | Reporting of having to self-advocate with providers, insurance, etc. |
| Sexual activity definition | How patient defines activity (e.g., penetration, touching) |
| Sexual activity importance/frequency | How important sexual activity was/is for patients. Includes frequency |
| Sexual activity important aspects | Aspects of sexual activity that were/are most important (e.g., penetration, orgasm, emotional, stress relief, intimacy) before cancer |
| Sexual function problems before | Any problems with function of sexual organs before cancer e.g., inability to achieve orgasm |
| Social worker | Mention of the importance or need for social worker |
| Support friends/family | Any mention of social support received/importance of social support from friends & family (excludes social worker or professional support/advice-this would be psych interventions) |
| Support groups | Any mention of support groups and peer support by cancer survivors. Includes strangers, excludes family/friends unless partner is part of support group |
| Support groups: desired content | Any description of the desired content of support groups |
| Support groups: desired frequency | Any description of the desired frequency of support groups |
| Support groups: disclosing sexual side effects | Any discussion of sexual side effects in support groups that patients participated in |
| Support groups: experience | Any experience with support groups after treatment |
| Support groups: helpful | Description of a specific way a support group was helpful (not just ‘it was great’) |
| Support groups: opening up | Reports opening up in support groups |
| Support groups: partner involvement | Description of partner involvement in support groups patient participated in (e.g., not involved, very involved) |
| Symptom reporting | Any reporting of side effects to provider |
| Symptom treatment | Any treatment for side effects (provider given or self-initiated) |
| Treatment options | Any mention of decision making related to treatment options for cancer or treatment of side effects |
| You know what I mean | Fillers such as "you know what I mean"/"you know", etc. (often a sign they are uncomfortable) |
