# Supplementary material for: Sexual activity and functioning after breast cancer treatment: perspectives on the importance of pleasure from a radiotherapy cohort
Source: BMC Womens Health. 2025 Oct 29;25:522. doi: 10.1186/s12905-025-04063-w (PMC12570742; doi:10.1186/s12905-025-04063-w)
Supplement: Supplementary file 2 — Supplementary Material 2. [file 12905_2025_4063_MOESM2_ESM.docx]

Qualitative data collection

Semi-structured Interview Guide

Note: the following semi-structured guide is intended to prompt spontaneous questioning and discussion based on interviewee responses, therefore all questions cannot be anticipated.

Thank you for taking the time to speak with me today. We are talking to people who have had breast or pelvic cancer to learn about the importance of sexual activity and sexual function, or how well your body parts work during sexual activity, before and after cancer treatment and to learn how treatment may have affected sexual function. There is a lot of scientific literature on sexual function in men after cancer treatment, but much less is known about women and gender-diverse patients and we are hoping to expand what we know. You and many others completed surveys on sexual health and this is an important first step in helping us build this knowledge. We are now talking to survivors to gather more detailed information to fully tell your stories. Here you are the teacher and we are learning from you. Please feel free to say anything you feel, we will not get offended. Our ultimate goal is to improve cancer care.

I would like to record this interview if you don’t mind so we can capture your words exactly. When I start the recording, I will try to avoid using your name so your information remains confidential. If I do use your name or if you mention any identifying information, we will remove it from the transcript so the data cannot be traced directly to you. Feel free to skip any questions or ask me to pause the recording if you would like to say something off the record. Do you have any questions before we begin? Is it okay for me to start the recording?

[click “live transcript”, and then “hide”]

< Start recording>

First, I am going to ask you some basic questions about how you define sexual activity. Then we will talk about sexual health *before* you were diagnosed with cancer. Then I’ll ask questions about *after* your diagnosis. And finally, we will talk about sexual health *after* treatment.

So first, I want to learn about how you define sexual activity.

1. What things go into the category ‘sexual activity’ for you?

Probes: intercourse, anal sex, oral sex, masturbation, petting/touching, type of activity, having a partner, ability to achieve orgasm

**Now I am going to ask you questions about sexual activity and sexual function before your cancer diagnosis.**

**When talking about sexual activity for the following questions, I’d like for you to consider not just vaginal intercourse but also other types of sexual activity to include any pleasurable and consensual sexual experiences, such as anal sex, oral sex, masturbation with or without a partner, or anything else you might consider sexual activity.**

**When talking about sexual function I mean how well your sexual organs are working. Sexual organs include the vulva or external genitalia, vagina, clitoris or erectile tissues, and nipples.**

1. Thinking back, how important was sexual activity to you *before* you were diagnosed with cancer or started to notice symptoms related to the cancer?
2. What aspects of sexual activity were most important to you?

Probe: for some people having an orgasm is important, for others, the emotional aspect is more important. What aspects were most important to you?

Follow up questions: timing/if issues were related to cancer diagnosis (bleeding/discharge, pain, fatigue) or menopause (desire/lubrication/discomfort/pain)].

1. *Before* you were diagnosed with cancer, what type of problems with sexual function did you experience? (Problems may include desire, discomfort/pain, lubrication, pleasure, ability to have orgasm)

4a. What type, for how long, any treatment?

1. *Before* you began treatment for your cancer, to what extent were you concerned about how treatment might affect your sexual health?

5a. What were you concerned about?

Probes: psychosocial and physical issues.

5b. Did you share your concerns with anyone?

Probes: Who? Response? How did it go? How did this make you feel?

1. *Before* your treatment, did anyone talk to you about how treatment might affect your sexual function?

Probes: Who (radiation oncologist, radiation team, other physician, other)? When? Did anything prompt? What was said? How did you perceive this information (welcome, helpful, embarrassed)? Was any effort made to minimize these side effects? (alternative therapies, pathways of radiation, treatments post radiation)

Do you think any aspects of your identity (gender, sexual ID, race/ethnicity) impacted that conversation?

1. What would have been helpful to know before you began treatment? Who would you want to have received this information from?

Probes: doctor, nurse, specific gender, same sexual identification, minority status? (e.g., only female physician?) In what format? Flyer, online materials, printout, video, in-person discussion.

**Now let’s focus on sexual activity and sexual function *after* you began cancer treatment.**

1. Describe the impact of cancer treatment on your sexual *activity.* [e.g., desire, frequency]

(Probe about severity and length of each issue)

1. Now let’s talk about how your body was functioning after treatment. Describe the impact of cancer treatment on how your sexual organs work? As I mentioned before, this can include the vulva or external genitalia, vagina, clitoris or erectile tissues, and nipples.

(Probe about severity and length of each issue).

1. Have you talked to anyone about the changes you have experienced? Who? What did they say? How did this make you feel? Any improvements? Still an issue?
2. How important is sexual activity to you now? What aspects are more important than others? (e.g., intercourse, orgasm, intimacy)
3. After cancer treatment some people use aids to prevent scarring or help with sexual activity. These may include personal lubricants, vaginal moisturizers, hormones such as pills or patches, vaginal dilators, vibrators, vacuum pumps, and vaginal rejuvenation. Tell me about your experience with sexual aids since your treatment. What barriers to using sexual aids have you faced? Probes: lack of knowledge/information, lack of access, pain, comfort level, time, personal space, partner support, religion.
4. Some people use psychological interventions, like relaxation, stress management, or mind-body interventions to help with sexual side effects of cancer treatment. What experience, if any, do you have with these types of treatment? Would you be open to these types of therapies? What would be feasible- online, in-person, interacting with an app? How many hours per month would be appropriate? Is there anyone else you’d want to include in the intervention (e.g., partner?). You mentioned (identity). How could someone personalize the treatment to better meet your needs or make you feel more comfortable?
5. Is there anything else that we didn’t discuss that you’d like to share?
